# Metabolic tumour volume as a prognostic factor for oral cavity squamous cell carcinoma treated with primary surgery

**DOI:** 10.1186/s40463-014-0033-7

**Published:** 2014-10-13

**Authors:** Han Zhang, Hadi Seikaly, Jonathan T Abele, Dean T Jeffery, Jeffrey R Harris, Daniel A O’Connell

**Affiliations:** Division of Otolaryngology-Head and Neck Surgery, University of Alberta, Edmonton, Alberta Canada; Department of Radiology, University of Alberta, Edmonton, Alberta Canada

**Keywords:** Metabolic tumour volume, Standardized uptake value, Imaging, Tumour marker, Head and neck cancer

## Abstract

**Background:**

Metabolic tumour volume (MTV) obtained from pre-treatment ^18^ F-fluorodeoxydeglucose positron emission tomography with computed tomography (PET-CT) has been validated as an independent predictive factor of outcomes in head and neck cancer patients (HNC) treated with primary chemoradiotherapy (CRT). However its role in patients treated with primary surgery has not yet been studied.

**Objective:**

To evaluate the prognostic value of MTV in patients treated with primary surgery for oral cavity squamous cell carcinoma (OCSCC).

**Method:**

Demographic and survival data was obtained from patients diagnosed with OCSCC from 2008–2012 in Alberta, Canada. All patients included in the study had PET-CT scan before curative surgical resection. MTV and maximum standardized uptake value (SUV_max_) value was delineated from pre-treatment PET-CT scans using Segami Oasis software (Columbus, OH). MTV and SUV_max_ were divided into intertertile thirds before statistical analysis to allow for in-group comparison of survival.

**Results:**

A total of 80 patients were analyzed using SPSS ver. 20.0 (SPSS Inc, Chicago, IL). Five-year overall, and disease-free survival using Kaplan-Meier curves were 70% and 73% respectively. When the combined SUV_max_ (tumour primary and locoregional metastasis) was evaluated, it failed to predict overall (HR = 1.0, p = 0.99) or disease-free survival (HR = 1.0, p = 0.227).

Conversely an increase in MTV of 17.5 mL (difference between the highest and lowest MTV tertile) was associated with a 12.4 fold increase in risk of disease recurrence (p < 0.001) and an 11.2 fold increase in the risk of death (p < 0.05).

**Conclusions:**

This study shows that MTV is an independent adverse prognostic factor for death and disease recurrence in OCSCC treated with primary surgery.

## Introduction

Head and Neck cancer (HNC) accounts for 5% of all malignancies worldwide with oral cavity squamous cell carcinoma (OCSCC) being the most common site of occurrence [1,2]. Traditionally, successful treatment and better prognosis often depended on tumour staging, local extension, anatomical tumour site, histotype, lymph node involvement and locoregional disease control [3-6]. Recent developments in tumour and protein markers have helped with better patient selection leading to improved survival benefits [4,5]. ^18^ F-fluorodeoxydeglucose positron emission tomography with computed tomography (PET-CT) plays a major role in the management of HNC patients owing largely to its ability to systematically measure tumour burden [7-10]. As a result it has become the standard of care in many centers for staging, evaluation of nodal and distant metastasis as well as key roles in radiation planning [7,10,11]. Maximum standardized uptake value (SUV_max_) delineated from pre-treatment PET-CT has had mixed results in its role as a prognostic factor for HNC treated with definitive chemoradiotherapy [4,12]. Metabolic tumour volume (MTV) calculated as the product of the SUV_max_ and tumour volume has recently been validated as an independent predictive factor of outcomes in head and neck cancer patients (HNC) treated with primary chemoradiotherapy (CRT) [5]. Its role in OCSCC treated with primary surgical resection (S) with the addition of radiotherapy (S-RT) and chemotherapy (S-CRT) has never been investigated.

The objective of this study was to assess the prognostic value of MTV in patients treated with primary surgery for OCSCC.

## Method

Ethics approval was granted by the University of Alberta’s Health Research Ethics Board (HREB) and the Alberta Cancer Board.

### Patients

Inclusion criteria were defined as:

1. Biopsy-proven OCSCC.
2. Treatment in Alberta with curative intent
3. Residents of Alberta > 18 years of age
4. PET-CT Scans prior to curative treatment

Exclusion criteria were defined as PET/CT

1. Previous HNC with or without treatment
2. Refusal of prescribed treatment
3. Treatment with palliative intent
4. Incomplete data sets from chart review

### Data collection

All patients diagnosed with an OCSCC within the Capital Health Zone in Alberta between January 1, 2008 and January 1, 2012 were identified in the Alberta Cancer Registry (ACR). The ACR, established in 1942, is a population-based registry that records and maintains data of all new cancer cases, their treatments, and resulting deaths occurring in the province. The ACR is operated by Alberta Health Services Cancer Care and follows patients longitudinally and prospectively [13].

Demographic, survival and clincopathologic data were extracted from the ACR database. A physical review of outpatient, inpatient, and cancer clinic records was undertaken to confirm data accuracy and extract relevant patient, tumour, treatment, follow-up, survival data, as well as Eastern Cooperative Oncology Group (ECOG) performance scores [14]. Charlson Comorbidity Index (CCI) scores, which were not included in the ACR database, were calculated using relevant comorbidities taken from chart review [15]. Date of diagnosis was defined as the date of pathologically confirmed OCSCC.

### Staging

Staging of the tumours was clinical and according to the seventh edition of the American Joint Committee on Cancer (AJCC) TNM staging manual [16].

### Treatment

All patients underwent curative surgical resection consisting of tumor ablation with variations of primary closure, locoregional or free tissue transfer reconstruction, and uni- or bilateral neck dissection. Patients receiving RT or CRT for distant metastases or palliation were not included. S-RT patients underwent surgical resection and adjuvant RT within 6–8 weeks post-operatively. S-CRT patients received surgical resection followed by adjuvant CRT within 6–8 weeks of their operation. Single agent cisplatin or carboplatin based CRT protocols were used exclusively for all patients.

### PET-CT imaging protocol

All patients were scanned using a Gemini TF 16-slice PET-CT scanner system (Philips Healthcare, Andover, MA). Patients were fasted a minimum of 4 hours before imaging (typically from midnight the night before). ^18^F-fluorodeoxyglucose (FDG) was injected intravenously at a dose of 5.18 MBq/kg. A 60-minute uptake period was waited prior to imaging.

#### PET imaging

With the patient in a supine position, PET scans were acquired in two separate acquisitions. Initially a scan was performed from the top of the shoulders to the mid-thigh level with the arms elevated. Scan times of 1 minute per bed acquisition were used for patients weighing less than 100 kg and 2 minutes for patients with a weight of 100 kg or greater. A second PET scan was performed from the mid brain level to the carina with the arms down at an acquisition time of 2 minutes per bed position.

#### CT imaging

After each PET images acquisition a corresponding CT scan was performed for both anatomic assessment and attenuation correction. This was typically performed with the administration of IV contrast. After the initial PET, 100 mL of intravenous contrast (Omnipaque 300, GE Healthcare, Buckinghamshire, UK) was injected at a rate of 3.5 mL/sec followed by 25 mL of saline at 3.0 mL/sec. A helical CT acquisition was obtained from the top of the shoulders to the mid-thigh level using dose modulation with a maximum 250 mAs, 120 kVp, and a contrast delay of 28 seconds. After the second PET acquisition a second IV contrast bolus of 40 mL at 1 mL/sec followed by 10 mL at 2.5 mL/sec followed by 25 mL saline at 2 mL/sec was administered. A second helical CT acquisition was then performed from the mid-brain to the carina using dose modulation with a maximum 350 mAs, 120 kVp, and 50 second contrast delay.

#### Image reconstruction

The PET images were reconstructed using vendor supplied ASTONISH time-of-flight iterative reconstruction with CT-based attenuation correction (Philips Healthcare, Andover, MA). The CT images through the neck were reconstructed for display with 1 mm slice thickness at 1 mm intervals. The CT images through the chest, abdomen, and pelvis were reconstructed for display with 3 mm slice thickness at 1.5 mm intervals.

#### Measurement of tumour volume

**Figure 1:**
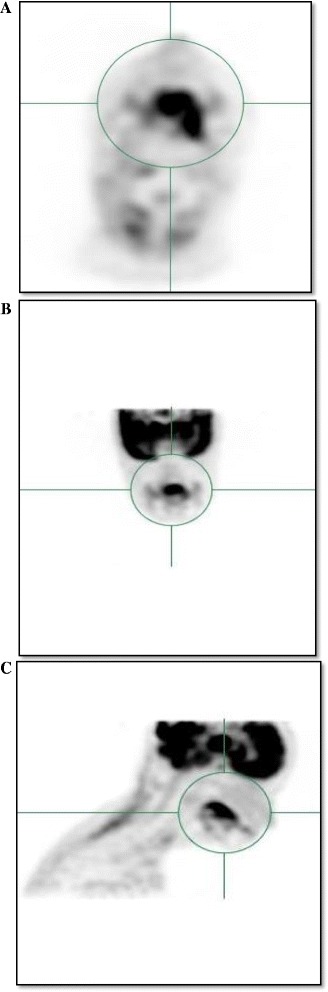
**Maximum intensity projection views of 18 F-fluorodeoxyglucose positron emission tomography scans for a patient with Stage III oral cavity cancer with (A) axial view, (B) coronal view, and (C) sagittal view.**

All images were reviewed using Oasis workstation software (Segami Corporation, Columbus, OH). The PET images, CT images, and fused PET-CT images were reviewed in transverse, sagittal, and coronal planes as show in Figure 1. A spherical volumetric region of interest was placed around the lesions of interest based on the evaluation of the PET and CT datasets. Diagnostic nuclear medicine reports and final radiation treatment planning volumes were used as reference when identifying the lesions. This was performed for the primary tumor and involved lymph nodes individually as well as the total group as a composite. MTV was defined as the volume of hypermetabolic tissue within the region of interest with a standardized uptake volume (SUV) greater than or equal to 2.5. This threshold intensity value has been identified and used in several previous studies validating MTV as a predictive factor for HNC [4,5]. This volume was automatically determined within the region of interest by the review software. The average SUV and SUV_max_ for the region with SUV greater than or equal to 2.5 was also determined.

### Outcomes

The primary outcome was set as the prognostic utility of MTV on overall survival. This was defined, as the time from the date of diagnosis to the date of death or last known date the patient was alive. Secondary outcome was set as the prognostic utility of MTV on disease-free survival, calculated as the time between the date of diagnosis to the date of OCSCC recurrence anywhere in the body. Thus, if patients died without any evidence of disease, they were considered disease free at the time of death.

### Follow-up

All patients were followed at regional cancer treatment centers at regular intervals following treatment. Dates of follow-up, up to November 1, 2013 were recorded. Patients who were suspected to have disease recurrence underwent a metastatic workup including appropriate imaging, endoscopy and biopsy as per standardized institutional guidelines.

### Statistical analysis

Baseline characteristics were compared using standard modes of comparison between multiple groups. Continuous data was analyzed using analysis of variance (ANOVA), with a Bonferoni correction factor for multiple comparisons. Categorical data was compared using the chi-squared test. Overall, disease-specific and disease-free survival rates were performed using Kaplan-Meier analyses to determine estimated actuarial survival rates. PET-CT endpoints (SUV_max_ and MTV) used for statistical analysis were the total MTV and SUV_max_ (value of the primary tumour in addition to any local region metastasis). Associations between PET-CT endpoints and outcome (disease-specific, disease-free, and overall survival) were assessed with a Cox proportional hazard model for univariate and multivariate predictions. Before entry into the proportional hazard model, each PET-CT endpoint was normalized to its intertertile ranges (difference between third and first tertile) to allow for in-group survival comparisons. Covariates of age, gender, clinical T-stage and N-Stage, ECOG performance status, and CCI scores were used for the Cox-regression analysis. Level of significance was set as p < 0.05. Analyses were performed with SPSS Statistics 20.0 (SPSS Inc, Chicago, IL).

## Results

One hundred and sixty-one patients were diagnosed with OCSCC in the capital health zone of Alberta from 2008 to 2012. Eighty-one were excluded from the final analysis: 11 refused any form of treatment, 5 had therapy outside of Alberta, 10 had palliative treatments, 45 patients did not receive a pre-treatment PET-CT. Five had misclassified tumour subsites, which were not in the oral cavity, and 5 had significantly incomplete data, precluding any data analysis. A total of 80 patients were left for analysis.

Table 1 demonstrates patient demographic and tumour variables. All patients included for analysis had undergone a standard metastatic workup and all were staged M0 at the time of diagnosis. The average age was found to be 62.1 (Range 30–87). The majority of the patient had cancer of the tongue (43.0%) and almost all had locally advanced OCSCC (15.0% Stage III and 53.8% Stage IV). Mean CCI and ECOG scores were found to be 1.2 (Range 0–6) and 1.0 (Range 0–3) respectively.

**Table 1:** **Characteristics of patients and tumors**

**Variable**	**Patients**
**n**	80
**Age**	
Mean, yrs	62.1
Range, yrs	30-87
**Gender, no. (%)**	
Male	47 (58.8)
Female	33 (41.2)
**Site of tumour, no. (%)**	
Tongue	34 (43.0)
Floor of mouth	15 (18.2)
Other	31 (38.8)
**cT-stage, no. (%)**	
1	4 (5.0)
2	26 (37.5)
3	18 (22.5)
4	32 (40.0)
**cN-stage, no. (%)**	
0	50 (62.5)
1	10 (12.5)
10	1 (1.0)
2b	9 (12.5)
2c	10 (12.5)
3	0 (0.0)
**Overall Stage (%)**	
I	4 (5.0)
II	21 (26.2)
III	12 (15.0)
IV	43 (53.8)
**Treatment, no. (%)**	
S	23 (28.8)
S-RT	37 (46.2)
S-CRT	20 (25)

*Abbreviations*: *no* number, *yrs* years, *cT-stage* clinical T-stage, *cN-stage* clinical N-stage, *S* surgery, *S-RT* surgery followed by adjuvant radiotherapy, *S-CRT* surgery followed by adjuvant chemoradiotherapy.

### Treatment analysis

Twenty-three (28.8%) patients received primary surgery as their only treatment modality and 57 (71.2%) patients had multi-modality treatment with the following classification: 37 (46.2%) had surgery followed by adjuvant radiotherapy and 20 (25%) patients received adjuvant chemoradiotherapy (Table 1).

### Survival analysis

**Figure 2:**
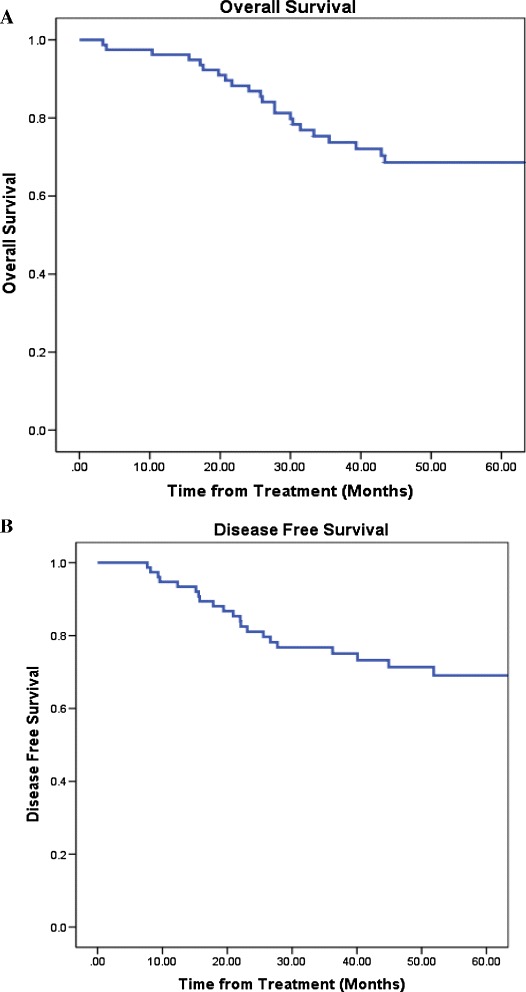
**Kaplan-Meier plots of (A) Overall survival, and (B) Disease-free survival rates for patients with oral cavity tumours.**

The mean follow-up for all patients was 1.9 year (SD = 0.9 years, Range = 0.3 - 4.2 years). Twenty-five patients died, with 19 succumbing to their OCSCC. Five patients died from non-head and neck cancer-related causes and 1 died of non-cancer related causes. Figure 2 demonstrates Kaplan-Meier for estimated overall and disease-free survival for all patients. Five-year overall and disease-free survival were found to be 70% and 73% respectively.

### Prognostic value

**Figure 3:**
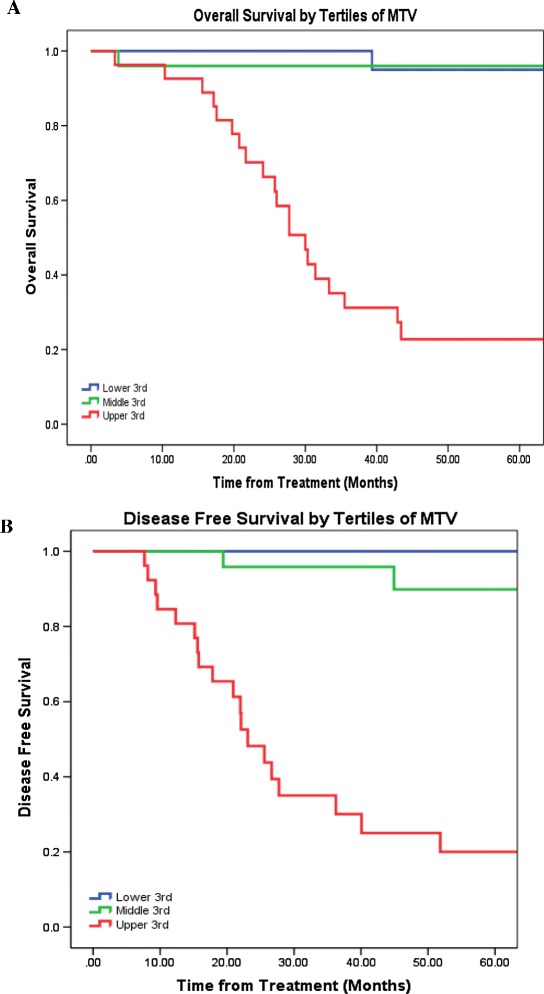
**Kaplan-Meier plots by Tertiles of Metabolic Tumour Volume (A) Overall survival, and (B) Disease-free survival rates for patients with oral cavity tumours.** Abbreviations: MTV, metabolic tumour volume.

MTV was defined as the MTV of the primary tumour in addition to any locoregional metastasis. The total MTV predicted disease-free and overall survival. The median total MTV was 7.4 cm^3^ (range, 2.1-72.1 cm^3^). Figure 3 shows overall and disease-free survival by tertiles of MTV. An After accounting for age, gender, CCI score, ECOG, T-stage, and N-stage, Cox-regression analysis showed an increase in MTV of 17.5 mL (difference between the first and third tertile) was associated with a 12.4 fold increase in risk of disease recurrence (p < 0.001) and a 11.2 fold increase in the risk of death (p < 0.05). When the combined SUVmax was evaluated, it failed to predict overall (HR = 1.0, p = 0.99) or disease-free survival (HR = 1.0, p = 0.227).

## Discussion

HNC diagnosis and treatment have evolved greatly in recent years [17-20]. The use of prognostic factors and tumour biomarkers in the past decade has changed the landscape of this debilitating disease [19,21]. Treatment outcomes however remain heterogeneous in nature. Substantial efforts have been focused on studying the identification of novel biologic parameters to further stratify risk groups with the goal of individualized treatment strategies for patients. PET-CT as an imaging modality has emerged as the standard of care in recent years for HNC patients aiding in staging, radiotherapy planning, and follow-up of treatment responses [8,11]. The ability of PET-CT to systematically measure tumor burden may directly predict locoregional control and survival as compared with previous identified clinical and pathological prognostic factors including age, anatomic subsite, and co-morbidity scores. MTV obtained from pre-treatment PET-CT as a variable of SUV_max_ and tumour size has been previously reported and validated to predict outcomes in HNC patients [4,5]. These results were only isolated to a group of patients treated with primary CRT, with the MTV showing an ability to predict treatment response to patients undergoing HNC treatments with CRT protocols. The impetus for this study was to examine the prognostic utility of MTV defined by ability to predict disease recurrence and disease specific survival from pre-treatment PET-CT in oral cavity squamous cell cancer (OCSCC) patients treated with primary surgery.

In this study MTV was shown to be an independent prognostic factor in a group of OCSCC patients treated with primary surgery. Figure 2 demonstrates patient overall and disease-free survival divided by tertiles. The difference in MTV between the highest MTV tertile and lowest was associated with a 12.4 fold increase in the risk of disease recurrence and 11.2 fold increase in the risk of death. This echoes the findings of La et al. who published the original paper on MTV as a prognostic factor HNC [4]. In their study, MTV was associated with a 1.9 fold increase in disease recurrence and 2.0 increase in the risk of death. The difference in results of our studies could certainly be attributed to our patient characteristics as well as our treatment modalities. Previously studies looking at MTV had a heterogeneous population consisting of multiple HNC subsites while our population was strictly OCSCC where the majority of patients had Stage IV disease. In addition, 25% of our patient population required triple modality treatment owing to the advanced nature of their disease. It is has been previously shown that S-CRT offers greater survival advantage given certain high-risk features on pathology as well as advanced tumour profiles [22,23]. Extracapsular spread, perineural invasion, as well as lymphovascular invasion could have certainly attributed to the results. These features were not studied independently, however as to their relationship with MTV in our study and remains to be explicated from further research.

AJCC TNM cancer staging system is used to classify tumours based on tumour dimensions as well as the presence of nodal disease. Traditionally, this staging system has been the standard of care in predicting outcomes in HNC. This cancer staging system, while addresses tumor variability does not account for gross tumour volume (GTV) [24]. Because many malignant lesions can be irregularly shaped or sized, this one-dimensional determinant is likely to fail to capture the information that a 3D approach can provide. GTV has been previously validated as a independent predictive factor for HNC outcomes [9]. The utility of GTV compared to the TNM staging system as a better predictor of survival outcomes remains to be elucidated after numerous studies [24,25]. In our study, T and N-staging was incorporated as part of a cox-regression analysis proving that MTV was an independent prognostic factor of OCSCC outcomes. However, it’s prognostic value in comparison to that of the TNM staging system was beyond the scope of the study. Further research looking into MTV versus that of the traditional tumor staging system could yield interesting results in the new era of radiologic imaging systems.

SUV_max_ is the most studied PET-CT metric for HNC. Predictive utility for SUV_max_ was initially promising however have been difficult to reproduce [12,26]. Moeller, et al. who conducted the first prospective trials on SUV_max_ was not able to show its reproducibility and validity as a prognostic factor for HNC treatments [27]. This lack of reproducibility was shown within several other studies looking at MTV and SUV_max_ utility as well. No study has previously looked at the relationship of SUV_max_ and primarily surgically treated HNC patients. While not the main focus of this study, SUV_max_ was not found to be a statistically significant prognostic biomarker for an OCSCC patient population treated with primary surgery. Certainly, further studies involving a prospective design could be utilized to help further clarify this relationship.

Limitations of this study are acknowledged. The population-based single institution design of the study precluded any controls. However, factors such as age, gender, T and N-stage, treatment modality, CCI as well as ECOG performance scores that could have been possible confounding effects were accounted for in a robust cox regression analysis. Our institution used a standardized protocol with PET imaging and a single software system for image analysis. Different imaging protocols, PET scanners, and imaging processing techniques would potentially affect the results and have an impact on the effectiveness of MTV. Given these limitations, a validation prospective multi-institutional study could be the next step in eliciting the relationship between MTV and survival in a primary surgically treated patient population.

The results of this study demonstrate utility of MTV as an independent and robust prognostic marker that is easily obtainable from pre-treatment PET-CT imaging in an OCSCC population treated with primary surgery. Overall survival as well as disease-free survival was both found be predictable by MTV. Previous studies that included patients treated with CRT have demonstrated its value in predicting outcomes. This is the first study, to our knowledge to report the use of MTV in patients with head and neck cancer treated surgically. Our findings suggest that MTV is not only a predictive factor for CRT treatments in HNC patients, but also an independent prognostic factor of overall and disease-free survival to patients with OCSCC. It is possible that MTV can be used in similar patient populations to provide guidance on types of adjuvant therapies for OCSCC.

## Conclusion

This population based cohort study demonstrates that pre-treatment MTV delineated from PET-CT is an independent and robust prognostic marker for OCSCC treated with primary surgery. Patients with higher MTV values should be further evaluated for the possibility of using escalated treatments regimens in their OCSCC treatment.

### Ethics approval

This study was approved by the University of Alberta’s Health Research Ethics Board.
